# Random Mutagenesis as a Promising Tool for Microalgal Strain Improvement towards Industrial Production

**DOI:** 10.3390/md20070440

**Published:** 2022-06-30

**Authors:** Mafalda Trovão, Lisa M. Schüler, Adriana Machado, Gabriel Bombo, Sofia Navalho, Ana Barros, Hugo Pereira, Joana Silva, Filomena Freitas, João Varela

**Affiliations:** 1 Allmicroalgae Natural Products S.A., R&D Department, Rua 25 de Abril s/n, 2445-413 Pataias, Portugal; mtd.santos@campus.fct.unl.pt (M.T.); adrisalom@gmail.com (A.M.); ana.barros@allmicroalgae.com (A.B.); joana.g.silva@allmicroalgae.com (J.S.); 2 GreenCoLab—Associação Oceano Verde, University of Algarve, Campus de Gambelas, 8005-139 Faro, Portugal; lisaschueler@greencolab.com (L.M.S.); gabrielbombo@greencolab.com (G.B.); sofianavalho@greencolab.com (S.N.); hugopereira@greencolab.com (H.P.); 3 Associate Laboratory i4HB—Institute for Health and Bioeconomy, School of Science and Technology, NOVA University Lisbon, 2829-516 Caparica, Portugal; a4406@fct.unl.pt; 4 UCIBIO—Applied Molecular Biosciences Unit, Department of Chemistry, School of Science and Technology, NOVA University Lisbon, 2829-516 Caparica, Portugal; 5 CCMAR—Centre of Marine Sciences, University of Algarve, Campus de Gambelas, 8005-139 Faro, Portugal

**Keywords:** reverse and forward genetics, selection methods, fluorescence-activated cell sorting, adaptive laboratory evolution, genetic engineering

## Abstract

Microalgae have become a promising novel and sustainable feedstock for meeting the rising demand for food and feed. However, microalgae-based products are currently hindered by high production costs. One major reason for this is that commonly cultivated wildtype strains do not possess the robustness and productivity required for successful industrial production. Several strain improvement technologies have been developed towards creating more stress tolerant and productive strains. While classical methods of forward genetics have been extensively used to determine gene function of randomly generated mutants, reverse genetics has been explored to generate specific mutations and target phenotypes. Site-directed mutagenesis can be accomplished by employing different gene editing tools, which enable the generation of tailor-made genotypes. Nevertheless, strategies promoting the selection of randomly generated mutants avoid the introduction of foreign genetic material. In this paper, we review different microalgal strain improvement approaches and their applications, with a primary focus on random mutagenesis. Current challenges hampering strain improvement, selection, and commercialization will be discussed. The combination of these approaches with high-throughput technologies, such as fluorescence-activated cell sorting, as tools to select the most promising mutants, will also be discussed.

## 1. Introduction

The world’s population is estimated to reach 9 billion people in 2050 [1,2], which raises significant concerns about the future energy, food, and feed demand, as well as waste management and dependency on limited resources [3,4,5]. In this context, microalgae are widely recognized as promising alternatives to conventional feedstocks and represent a potential part of the solution to address these worldwide issues.

Microalgae are the major contributors to CO_2_ fixation and O_2_ production across the globe, with the ability not only to mitigate the rising CO_2_ levels, but also to utilize nutrients from effluents that otherwise would be discharged into the environment. Thus, the production of microalgae makes a large contribution to the field of bioremediation and leveraging circular economy [2,6,7,8]. In addition, microalgae might play a key role as part of alternative feedstocks to face global food and feed scarcity, since they do not require arable land to be cultivated and possess very rich nutritional profiles, being a source of proteins, lipids, carbohydrates, polyunsaturated fatty acids (PUFAs), vitamins, and bioactive compounds [9,10,11,12].

Despite the outstanding potential of microalgae for different biotechnological applications, the commercialization of microalgae-based products, such as the biomass itself or added-value compounds (e.g., pigments and PUFAs), is still restricted to high-value niche markets. Current high production costs and unsuccessful attempts at marketing and selling these products has hindered the development of the microalgal industry and market [6,13]. Concerning cultivation, the prevailing bottlenecks include low biomass conversion efficiencies, low target biocompound productivities, low light delivery in concentrated cultures under photoautotrophic cultivation, wide environmental variations under outdoor conditions, contamination of cultures, and costly inputs, namely culture media and energy demand [14,15,16,17]. Open systems arose as cheaper cultivation systems, as compared to closed systems. Although the former have enabled a reduction in energy costs [18,19], they are prone to contamination, and if the microalga is not robust enough to outgrow grazers and competitors, culture crashes generally ensue. Moreover, the exposure to abiotic stress factors (e.g., temperature and salinity) might impact overall productivity, unless a robust and stress-tolerant strain is used [19]. Additionally, downstream processing usually implies high energy consumption with concomitant high operating expenses (OPEX), often with a low recovery of biomass and production surpluses [14,20,21,22].

Overcoming these hurdles requires a multistage optimization approach in the whole microalgae production and processing pipeline, to tackle the current bottlenecks of industrial-scale cultivation of microalgal biomass [14]. Rethinking and revamping the whole pipeline under a biorefinery and circular economy approach would contribute to more economically feasible industrial-scale processes [12,23,24]. On the other hand, strain selection and improvement are crucial stages in generating industrial strains and facilitating large-scale microalgal production [13,23,25]. However, this area has not received enough attention and a more effective research effort is still required.

Currently, only a few naturally occurring microalgal strains meet the required traits for economically viable industrial production for use in diverse biotechnological applications [6,26]. Thereby, it is important to isolate and create novel strains able to face the challenges mentioned above. The first step towards this purpose begins with bioprospection and selection of strains with improved features and enhanced biomass and target biomolecule productivities. High-throughput technologies, such as methods based on fluorescence-activated cell sorting (FACS), are powerful tools to mine and isolate improved strains [13,27,28,29]. Nonetheless, to pursue large-scale production and profitability, such strains require further improvement.

Traditional industries, such as agriculture, cattle farming and even pharmaceutical companies, have undergone significant development through investing in breeding as well as in random mutagenesis and targeting strategies, such as the generation of genetically modified organisms (GMOs) to create more resilient and productive strains rather than using their wildtype counterparts [5,30,31,32].

Classical forward genetic studies with microalgae allows the identification of genes, the assignment of phenotypes to a gene sequence/genotype and the expansion of our current understanding of the biology and metabolism of several microalgal species [13,33,34]. Throughout the years, this acquired knowledge has shed light on metabolic pathways and genotypes, which are now used by the scientific community to perform reverse genetics experiments that target specific gene sequences to improve microalgal strains and generate novel phenotypes [34,35].

Genetic variability and species evolution occurs naturally and randomly by exposure to UV irradiation from sunlight, reactive oxygen species (ROS) or other agents that cause spontaneous mutations in the genetic material [35,36,37,38,39]. Since these processes are slow and untargeted, strategies, such as random mutagenesis and adaptive laboratory evolution, have been applied to accelerate these naturally occurring processes, which enable the generation and the selection of mutant organisms with properties that meet the needs of industry [2,31,35]. A different strategy used to improve the key characteristics of microalgae is the promotion of site-directed mutagenesis by resorting to gene-editing tools, such as clustered regularly interspaced short palindromic repeats (CRISPR)/CRISPR-associated protein 9 (CRISPR-Cas9) and zinc finger nucleases (ZFN). Moreover, RNA interference (RNAi), together with progression in the synthetic biology field, has been used to develop tailor-made genotypes by targeting specific genes to increase both the biomass and the yields of added-value compounds, thereby reducing production costs [2].

As several technologies have been applied to microalgal strain improvement to overcome the main hindrances of microalgal production, diverse overviews of this topic have been published. Spicer and Molnar (2018) have published a useful discussion about gene editing in microalgae and its correspondent challenges and perspectives [32]. Other review papers have reported advances regarding the application of adaptive laboratory evolution [8,40,41], high-throughput techniques, and genetic engineering to microalgae [2,6,7,13,42]. In turn, Aklilu (2021), Torres-Tiji et al. (2020), and Hlavova et al. (2015) provided a general overview on the importance of microalgal strain improvement and the different technologies available [5,34,35].

In this review, different strain improvement approaches will be discussed and compared, focusing on the current pipelines that combine random mutagenesis with high-throughput technologies, based on the combined use of FACS and metabolic inhibition, as a tool to improve microalgal strains for industrial purposes. Specific case studies as well as the advantages and disadvantages of using such pipelines will be discussed. Future perspectives for fast-tracking the improvement of microalgal strains will also be provided. Lastly, the current regulatory frameworks on GMOs applied to microalgae will be summarized and discussed.

## 2. Strategies for Microalgal Strain Improvement

**Figure 1:**
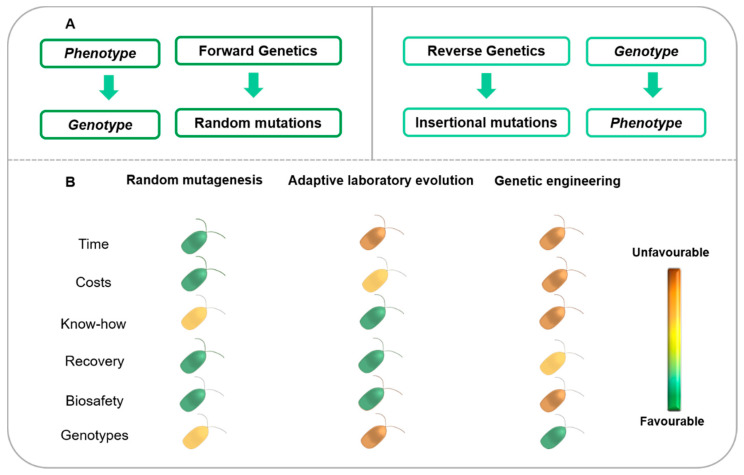
Strain improvement approaches by forward and reverse genetics strategies (**A**). Comparison of several aspects of three methods of strain improvement: random mutagenesis, adaptive laboratory evolution and genetic engineering (**B**). Time—time required to perform the experiments and obtain results; Costs—general costs of using these methods; Know-how—level of knowledge required to implement the technology; Recovery—ease of selection and isolation of strains with the desired features; Biosafety—potential biosafety concerns for consumers and environment over the strains obtained; Genotypes—ability to attain the desired genotypes and phenotypes.

Classical genetics, also known as forward genetics, is based on the observation of phenotypes and the identification of the gene sequences responsible for specific phenotypic features, within which, diversity might arise through naturally occurring or induced mutations [34,43]. In forward genetics, the target phenotype is selected, while the genotype is unknown (Figure 1A) [35]. Conversely, reverse genetics starts by introducing alterations in a known gene sequence through random or site-directed mutations, which are then translated into an observable phenotypic change, elucidating the respective function of the gene or set of genes (Figure 1A) [35,43,44]. Over recent years, whole genomes have been sequenced; this has resulted in an increased focus on reverse genetics, since scientists are now able to change or even disrupt specific target genes in order to observe and study the effects of these alterations on the phenotype [34].

Forward genetics is also particularly useful, since it comprises tools such as random mutagenesis and adaptive laboratory evolution, which allow the generation of large pools of mutant phenotypes without any previous knowledge about the genetics and metabolism of the target organism and without the need for the development of molecular tools, which can be time-consuming and more expensive than the application of random mutagenesis and adaptive laboratory evolution strategies (Figure 1B) [5]. The most desired features, namely higher biomass and target compound productivities as well as higher tolerance to specific growth conditions, are selected first, while the respective mutation is identified afterwards [34,35,43].

Random mutagenesis is a robust, well-established, easy-to-perform and cost-effective tool used to generate mutants (Figure 1B) [45,46]. As mentioned above, random mutagenesis is merely an acceleration of naturally occurring processes achieved by exposing organisms to a potent physical or chemical mutagenic agent followed by the selection of mutants with the desired features [11,13,31,35]. The mutations are non-specific; however, there is no introduction of foreign genetic material, which allows for the production of these mutants at industrial facilities without the restrictions imposed on GMOs that contain heterologous DNA sequences [5,11,31,46]. Although a great variety of mutants can be generated by random mutagenesis, the mutations are often lethal or at least detrimental to the mutagenized organisms. In addition, mutant characteristics can also be unstable and reversible, which highlights the importance of developing effective selection methods and ensuring phenotypic stability. Indeed, the lack of adequate selection methods is the most significant limitation of this technology, which has been addressed through the use of high-throughput methods, such as FACS, as discussed below in this review [13,25,45,47].

Adaptive laboratory evolution is another cost-effective approach that does not require previous knowledge of the genetics of the microalgal strain under investigation (Figure 1B). Furthermore, it also avoids the introduction of foreign genetic material into the target cells; thus, the improved strains pose no regulatory issues due to the long biosafety record of such technologies (Figure 1B) [48,49]. In adaptive laboratory evolution experiments, cell cultures are subjected to a continuous selective pressure over a long period of time [25,40,41,50]. Consequently, selection can act on all traits responsible for a suitable improvement under the environmental regimes of interest [25,40,41,49,51]. Thus, spontaneous, adaptive and non-specific mutations are introduced into the genome and passed down from mother to daughter cells [41,51,52]. However, adaptive laboratory evolution is a highly laborious and time-consuming strategy that requires many generations to obtain the desired phenotype (Figure 1B) [50].

As an alternative technique to forward genetics, insertional mutagenesis has also been used to generate and select microalgal mutants of interest. In contrast with random mutagenesis and adaptive laboratory evolution, this technology requires the availability of a DNA transformation protocol for each organism and exogenous DNA is introduced via insertional mutagenesis. This approach places DNA fragments in coding or non-coding regions of the genome, promoting gene disruption and/or insertion and thus, yielding novel mutants, in which the mutation will be more easily identified by the presence of selective markers and/or genetic (e.g., unique sequences) or phenotypic (e.g., expression of fluorescent gene products) tags [25,50,53,54,55]. This approach has the advantage of facilitating the identification of the mutation site associated with a specific mutant phenotype. However, unlike random mutagenesis and adaptive laboratory evolution, insertional mutagenesis has the disadvantage of producing GMOs via the introduction of foreign DNA.

Regarding reverse genetics methodologies, once the genotype is available, most include the introduction of foreign genetic material into cells, rendering them GMOs, and consequently, bringing forth several concerns and commercialization hurdles, as discussed below (Figure 1B) [32,50,56,57]. However, genetic engineering allows the generation of tailor-made genotypes with specific mutations in the genes of interest, which may affect their sequence and/or expression, usually leading to a loss-of-function mutation through, for example, gene silencing via RNA interference (RNAi) [34,35,43,44,58]. Engineered nuclease systems, namely ZFN, transcription activator-like effector nucleases (TALENs) and CRISPR-Cas9 are versatile tools that create double-strand breaks by cleaving DNA, which enable knockin mutations through the insertion of an intervening DNA fragment or knockout/knockdown mutations via deletion or changes in the nucleotide sequence. These strategies can either enhance or impair gene expression and add or delete genes [13,50,59,60,61,62,63,64]. However, applying these gene-editing techniques requires a priori knowledge of the target organism’s genome (Figure 1B), but not of the gene function; hence, it enables the analysis of the effects of such mutations on the phenotype [35]. Alternatively, unspecific gene disruption can be promoted to generate mutant phenotypes, for example, by targeting-induced local lesions in genomes (TILLING) [34,35,43]. TILLING has the advantage of not introducing foreign genetic material, since it consists of finding mutations in target genes with heteroduplex formations through the use of an endonuclease that specifically cleaves ethyl methanesulfonate (EMS)-induced mismatches [43,65,66]. Basically, this technique involves EMS-induced chemical mutagenesis followed by high-throughput screening for point mutations [65]. Despite the potential specificity of the site-directed approaches described earlier, defining a specific genomic target is difficult. A given alteration in the genome often has pleiotropic effects on a wide set of genes, which may not only hinder the isolation of mutants with the desired phenotypes but may also impair their viability and/or growth patterns (Figure 1B). Moreover, molecular biology models and transformation protocols are available for only a few microalgal species (e.g., *Chlamydomonas reinhardtii*). As a result, efficient molecular and transformation tools need to be developed for other species; thus, this is a time-consuming and potentially expensive strain improvement approach (Figure 1B) [13,44,54,62].

Major factors leading to failure in microalgae production and in the commercialization of their biomass and bioproducts, have been addressed by each strain improvement strategy described above. One such factor is the lack of robustness and tolerance of environmental abiotic factors and cultivation conditions, in particular temperature, salinity, shear stress, and exposure to toxic compounds and pollutants. In the following sections, we provide examples of the application of strain improvement strategies for improving biomass productivity and enhancing yields of target compounds (e.g., triacylglycerols, PUFAs, and pigments). Each strain improvement approach might be used to achieve different goals, and so their respective benefits, concerns, and restrictions (summarized in Figure 1) will also be discussed.

### 2.1. Random Mutagenesis

#### 2.1.1. Historical Perspective

Plant and animal wildtype strains have seldom been considered suitable for large-scale production [67]. Over the years, mankind has tried to improve the robustness, productivity, and nutritional value of plants, animals, and microorganisms for food and feed production [32]. Early in the 20th century, geneticists and biologists were interested in gene mutations and heredity, acknowledging them as the basis of the evolution of life [68]. However, naturally occurring mutations are rare events, and are difficult to detect and study [68]. The first immediate approach to altering natural organisms for the benefit of humans was carried out by breeders who started to recombine different genetic materials to obtain new strains that combined the features of both parental organisms, and selecting the best-performing individuals [32,68]. Nevertheless, scientists were eager to go beyond naturally occurring mutations and breeding. The discovery of random mutagenesis refers back to 1921, when Mavor first demonstrated that X-rays had a mutating effect on *Drosophila melanogaster* chromosomes [69]; this was followed by Little and Bagg (1924), whose work corroborated this effect of X-rays on mice [70].

The discovery of mutagenic agents, such as X-rays, led to an experimental revolution in genetics, since researchers could now partially control mutagenesis to generate mutant progenies [71]. In the 1950s, random mutations in microalgae were also studied in order to understand pigment biosynthesis [72]. Other genetic agents, and their mutagenic properties, began to be studied, namely chemical mutagens [73]. Reports of one of the first microalgal mutants refer back to 1960, when Schwarze and Frandsen obtained a colorless *Chlorella* mutant through exposure to radioactive isotopes [74,75]. EMS and nitrosoguanidine (MNNG) also began to be studied as mutagens applied to algae [76,77]. At this time, EMS became, and remains to this day, one of the most frequently used mutagenic agents [73].

Over the past 100 years, random mutagenesis has been used as an easy-to-perform and robust tool to develop mutants based on a phenotype-driven search instead of focusing on specific gene modifications [45,46,78]. Random mutagenesis has received increased attention in the microalgae field, as it has been recognized as a very useful approach for creating more productive strains, regarding biomass and target compounds, and for adapting strains to tolerate a wider range of environmental conditions, with the advantage of not requiring extensive knowledge of microalgal genetics [6,46,47,67]. Similar to adaptive laboratory evolution, unspecific mutagenic action targets a set of genes simultaneously, which, with suitable selection methods, can be used to readily isolate strains associated with the intended phenotype, at a much faster rate [67,78]. Furthermore, since both chemical and physical mutagens are well-characterized, it is a ready-to-use technology that produces rapid results [35,45,79]. However, mutations are often deadly or hamper growth, and can revert to the wildtype over time, which hinders the isolation of stable mutant strains [47,80]. Strategies to prevent phenotypic reversion should be studied to enable the persistence of the improved mutants. In addition, a beneficial phenotype can only be isolated if it is possible to select it [47]; thus, more selection methods are required to enable the isolation of different mutants, namely, by resorting to high-throughput screening methods, as indicated below.

#### 2.1.2. Physical and Chemical Mutagenesis

**Figure 2:**
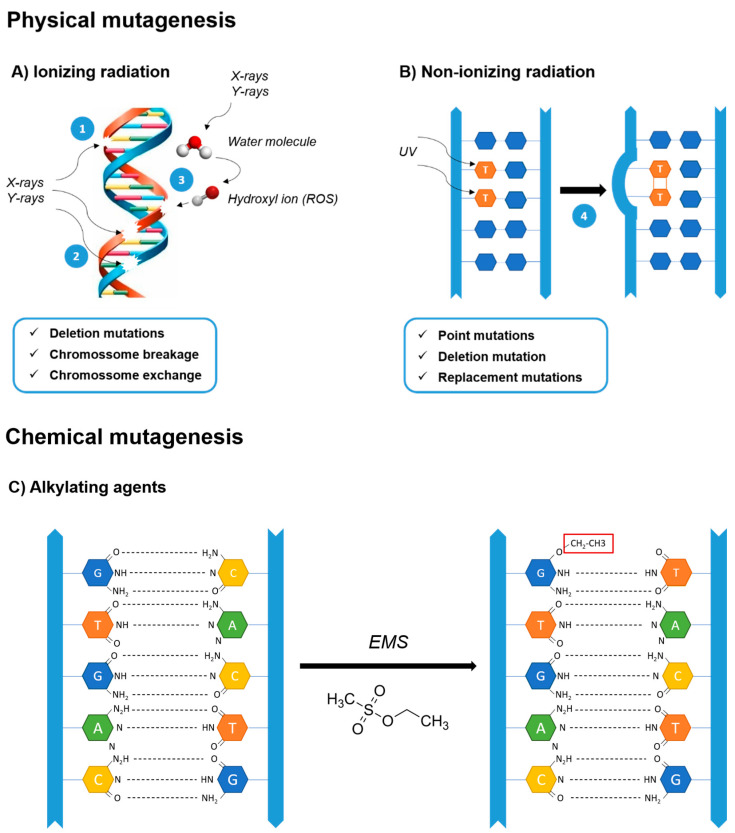
DNA mutation mechanisms by physical and chemical mutagenesis. (**A**) Ionizing radiation may induce the following lesions on DNA: 1—single-strand breakage; 2—double-strand breakage; and 3—reactive oxygen species (ROS) damage. (**B**) Non-ionizing radiation might cause: 4—thymine dimerization (DNA kink). (**C**) Alkylating agents, such as EMS, replace a hydrogen ion with an alkyl group on a DNA base, often guanine (G).

Random mutagenesis is carried out by treating a cell culture with a mutagen that usually induces single-nucleotide changes or small deletions in the genome that might encompass multiple genes or regulatory sequences [32,78]. Both chemical and physical mutagenic agents can be used and will act on the genome in different ways (Figure 2). Upon mutagenesis, strain selection is carried out depending on the target phenotype. However, these mutagenic agents are dangerous to work with; contact should be avoided and precautions must be taken to handle them. For example, chemical toxic agents should be handled in a fume chamber [79,81,82].

Physical mutagenesis consists of applying specific dosages of radiation to cells by means of ultraviolet (UV), laser, X-ray, heavy-ion and gamma irradiation [6,45,79,83,84,85]. Atmospheric and room temperature plasma (ARTP) mutagenesis is a more recent method that uses room temperature plasma to generate mutations, but also chemical species that might be mutagenic, being thus a possible physicochemical method [86].

Gamma and heavy-ion beam irradiation are both forms of high-frequency radiation that cause double-stranded DNA breakage by ionization, which often leads to the deletion of nucleotides as well as chromosome breaks and exchanges, respectively (Figure 2) [84,87,88]. Their higher frequency enables stronger cell penetration, which, by interacting with molecules such as water, gives origin to free radicals, which are able to disrupt macromolecules, namely DNA, causing high mutation rates [78,83]. Both methods, along with others, such as laser mutagenesis, involve the application of electromagnetic fields to mechanically induce changes in the DNA; however, these procedures usually require sophisticated equipment [85]. Therefore, UV radiation-mediated mutagenesis is often more appealing, since it is simpler, less expensive and easier to apply; it basically consists of exposing cells to UV sterilizing lamps commonly found in flow chambers, a basic piece of equipment available in most laboratories [89]. Moreover, it facilitates the isolation of mutants in sterile conditions, which often prevents the occurrence of biological contaminants [89]. Despite its lower frequency and lower mutation rate, UV radiation usually induces point mutations, deletions and replacements [90,91]. The underlying mechanism causing these mutations is based on UV absorption by DNA molecules, which leads to covalent linkage of pyrimidines, forming dimers that prevent normal base pairing and distort the DNA double-helix structure (Figure 2) [92,93]. Likewise, normal base pairing and double helix unwinding for replication and transcription cannot occur, resulting in a wide range of mutations [35,78,90]. Nonetheless, UV mutations are more prone to reversion and impermanence due to the existence of several UV damage repair mechanisms. To cope with the most common lesions induced by UV radiation, cyclobutane pyrimidine dimers, cells carry out nucleotide excision repair, which replaces lesion sites with newly synthesized oligonucleotides [94,95,96,97].

Chemical mutagens have also been widely used, and their mutagenic potential as well as their mechanisms of action are well-characterized [79,98]. The most commonly used chemical mutagens are alkylating agents, i.e., molecules that carry an active alkyl group, which substitutes a hydrogen ion for an alkyl group on a DNA base, often guanine (Figure 2) [6,45,79,99,100]. Upon DNA replication, nucleotide substitutions, insertions or deletions are introduced into the DNA sequence, often due to the misreading of the nucleotides on the chemically altered template strand by the DNA polymerase. The common alkylating agents are ethyl methanesulfonate (EMS), methyl methanesulfonate (MMS), nitrosoguanidine (NTG, MNNG), ethyl-nitrosourea (ENU) and *N*-methyl-*N*-nitrosourea (MNU), whereby EMS and NTG/MNNG are most frequently used in microalgal strain improvement [82,90,93]. These agents trigger a similar chemical mutagenesis mechanism in DNA, which enables high-frequency point mutation and the emergence of novel phenotypes [46,78,87,98]. However, EMS alkylation is specific to guanine, resulting in G/C to A/T transitions, while MNNG induces a wider spectrum of mutations [46,87,101,102].

#### 2.1.3. Mutant Selection Methods

**Figure 3:**
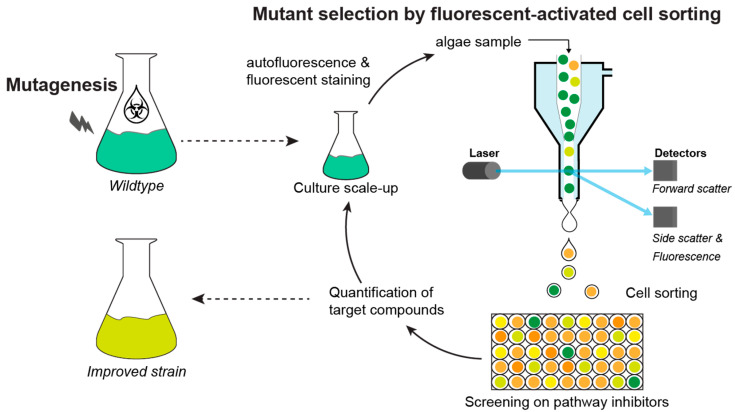
Random mutagenesis and high-throughput mutant selection pipeline using fluorescent-activated cell sorting (FACS) and pathway inhibitor screening.

The result of random mutagenesis is the generation of hundreds of mutant colonies. However, only a minute portion of them has the desired phenotype; thus, an efficient screening method is often necessary (Figure 3). Mutants can be selected via different properties, such as visual appearance, autofluorescence, or growth performance measured by absorbance. The large sizes and different colors of the colonies are good indicators for fast-growing mutant strains as well as changes in pigment contents, respectively. On the other hand, the autofluorescence of pigments, such as chlorophyll and carotenoids, measured by fluorescence imaging provides a good selection tool for differently pigmented mutants, e.g., truncated antenna size mutants [103]. Nevertheless, these screening techniques are very time-consuming, as each colony needs to be inspected individually and do not necessarily lead to the desired improved strain.

A more direct approach is the exposure of newly generated mutants to environmental stresses, such as extreme salinities or temperatures, light or dark conditions, CO_2_ levels, or nutrient stress. For example, a yellow mutant of *Chlorella vulgaris* was isolated upon mutagenesis followed by growth in the dark; mutagenesis was crucial to suppress the need for energy supply via photosynthesis, and thus, chlorophyll synthesis (Table 1) [11]. Furthermore, the exposure of mutagenized cells to high salinity, high temperature, or high pH followed by the selection of large colonies led to the generation of salt-resistant, thermotolerant and alkali-tolerant strains of *Chlorella* sp., respectively (Table 1 and Table S1) [104,105,106,107,108].

The most frequently used and selective process of mutant screening for high compound accumulation is the utilization of pathway inhibitors that specifically target rate-limiting enzymes of the biosynthesis of the desired compounds (Figure 3). Upon mutagenesis, colonies resistant to these inhibitors often contain mutations in the gene encoding an enzyme or a regulatory factor of the (partially) suppressed metabolic pathway. These mutations often cause higher metabolic flows through enhanced gene expression to overcome the metabolic inhibitor during the selection procedure [116]. In turn, such changes often lead to higher compound content and/or productivities in the respective mutants.

Carotenoid hyperproducing mutants can thus be isolated by the screening of pathway inhibitors, such as norflurazon, fluoridone, nicotine and diphenylamine, that block carotenoid biosynthesis (Table S1). More specifically, norflurazon and fluoridone inhibit phytoene desaturase, which is responsible for the desaturation of phytoene to phytofluene [117]. However, norflurazon-resistant mutants of *Tetraselmis striata* did not only show higher carotenoid content but also higher eicosapentaenoic acid (EPA) content, which suggests that norflurazon may also block the fatty acid desaturases of the PUFA pathway [116]. A similar pleiotropic effect has been found for diphenylamine (DPA), which is also widely used as an inhibitor of phytoene desaturase; however, in *Haematococcus pluvialis*, DPA-induced inhibition of *β*-carotene oxygenation and hydroxylation has been described, two key steps for the production of the xanthophyll astaxanthin [118,119]. To further enhance the levels of carotenoids, nicotine-induced blockage of lycopene cyclase can be used to isolate mutants able to overcome the inhibition of lycopene cyclization into *β*-carotene [120].

When lipid or fatty acid contents are the target of improvement, inhibitors such as cerulenin, quizalofop or erythromycin can be applied. Cerulenin is known to inhibit the *β*-ketoacyl-(acyl carrier protein) synthase I [121], while quizalofop inhibits the acetyl-CoA carboxylase (ACCase), both leading to alterations in fatty acid biosynthesis [122]. On the other hand, erythromycin is an antibiotic, targeting the protein synthesis of bacteria, but has been shown to affect chloroplast metabolism in microalgae by inhibiting the photosynthetic electron transport chain, leading to the damage of the photosystems and decreased pigment biosynthesis [123]. Nevertheless, Chaturvedi and Fujita [124] developed an erythromycin-resistant mutant of *Nannochloropsis oculata*, yielding increased contents of EPA (Table S1).

A different pathway of interest is the synthesis of sterols, which can be blocked by the herbicide terbinafine, which inhibits the enzyme squalene epoxidase. Upon mutagenesis, terbinafine-resistant mutants of *C. reinhardtii*, that overproduced sterols and squalene without compromising growth performance, were isolated (Table 1) [112].

Instead of targeting pathways specific to the synthesis of the biomolecules chosen for improvement, the focus can be on general metabolic fluxes. For example, ammonia assimilation can be inhibited by glufosinate, which blocks the essential enzyme glutamine synthetase. In this way, a metabolic condition similar to nitrogen starvation is triggered, which is a known inducer of lipids and certain carotenoids. Glufosinate-resistant mutants of *Haematococcus pluvialis* and *Coelastrum* sp. with higher astaxanthin contents than the wildtype have been isolated (Table 1 and Table S1) [99,110].

Taken together, the utilization of pathway inhibitor screening has led to promising mutants with improved biochemical profiles in different types of microalgae; however, these inhibitors often have a pleiotropic effect on overall metabolism and may lead to unexpected or unwanted mutants.

Another approach to mutant screening is the selection of desired traits by high-throughput methods such as FACS (Figure 3). A key characteristic of microalgae is the autofluorescence of several pigments, such as the wine-red fluorescence of chlorophyll *a* or the carotenoid fluorescence in the green range of the electromagnetic spectrum [125,126]. Furthermore, lipids can be stained by fluorescent dyes such as Nile red or BODIPY505/515. Upon random mutagenesis, lipid- or carotenoid-rich mutants have been isolated by the high-throughput selection of target cells via FACS [6]. Nevertheless, the difficulty in this method is the need for fluorescence, which is only displayed by pigments, solvatochromic dyes, and other signal-specific fluorochromes. Therefore, the correlation of fluorescence to certain compounds needs to be established. For example, in a study on *Phaeodactylum tricornutum*, the correlation between fucoxanthin and chlorophyll autofluorescence was used to isolate high-fucoxanthin-producing mutants (Table 1) [88].

#### 2.1.4. Random Mutagenesis Applications

One of the most important targets in strain improvement is growth performance and the resulting biomass volumetric productivity. Since the evolution of microalgae occurred under light-limiting conditions, microalgae possess increased contents of chlorophyll molecules and large chlorophyll antenna to maximize light utilization [127]. However, under photoautotrophic cultivation, growth performance depends heavily on a sufficient light supply and the self-shading effects of highly concentrated cultures often limit cell growth at an industrial scale. To improve light distribution in the reactor, mutants with lower chlorophyll contents and/or truncated antenna size are of interest. These often pale-green mutants have been isolated from *Chlorella vulgaris*, *Chlorella saccharophila*, *Chlorella sorokiniana*, *Nannochloropsis gaditana*, *Cyclotella* sp. and *Chlamydomonas reinhardtii* upon EMS- or UV-induced mutagenesis (Table 1 and Table S1) [55,103,111,128,129,130,131].

In recent years, researchers have also aimed for more appealing biochemical profiles with increased contents of a group of biomolecules (e.g., lipids and protein) or higher yields of added-value target compounds (e.g., pigments and/or PUFAs), depending on the species. Upon UV radiation-mediated mutagenesis, followed by screening using iodine vapor staining, starchless mutants of *Tetradesmus obliquus* (syn. *Scenedesmus obliquus*) with 41% increment in total fatty acid (TFA) productivity were isolated (Table 1) [114]. Conversely, Zhang et al. [98] used EMS-induced mutagenesis and Nile red fluorescence-based screening to isolate high-lipid-producing *Desmodesmus* sp. mutants (Table 1). A similar approach was also used for other species, such as *Nannochloropsis gaditana, Nannochloropsis oceanica* and *C. reinhardtii* (Table S1) [132,133,134]. Furthermore, a FACS-based selection using BODIPY staining showed success in the isolation of high-fatty-acid-producing mutants of *Microchloropsis salina* (syn. *Nannochloropsis salina*) and *Chlorella* sp. upon mutagenesis with EMS (Table 1) [109,135]. Moreover, Sarayloo et al. [115] used a combination of UV radiation and EMS to mutate *C. vulgaris* and its isolated mutants exhibited 67% increased lipid content and 35% increased biomass than those of the wildtype (Table 1). In a different study, physically induced random mutagenesis using gamma rays led to *Chlorella* mutants suitable for biodiesel production (Table 1) [113].

Tolerance of unfavorable environmental conditions can also be improved through random mutagenesis approaches. For example, Ong et al. [104] and Sachdeva et al. [136] managed to create thermotolerant mutants of *Chlorella* sp. through random mutagenesis with EMS, which allowed them to improve its growth rate by 1.8–6.7-fold at temperatures ranging between 25–40 °C (Table 1 and Table S1). On the other hand, NTG-induced mutagenesis combined with a screening of large colonies on pH 11.5 agar plates led to the isolation of alkali-tolerant *Chlorella* strains [106].

**Figure 4:**
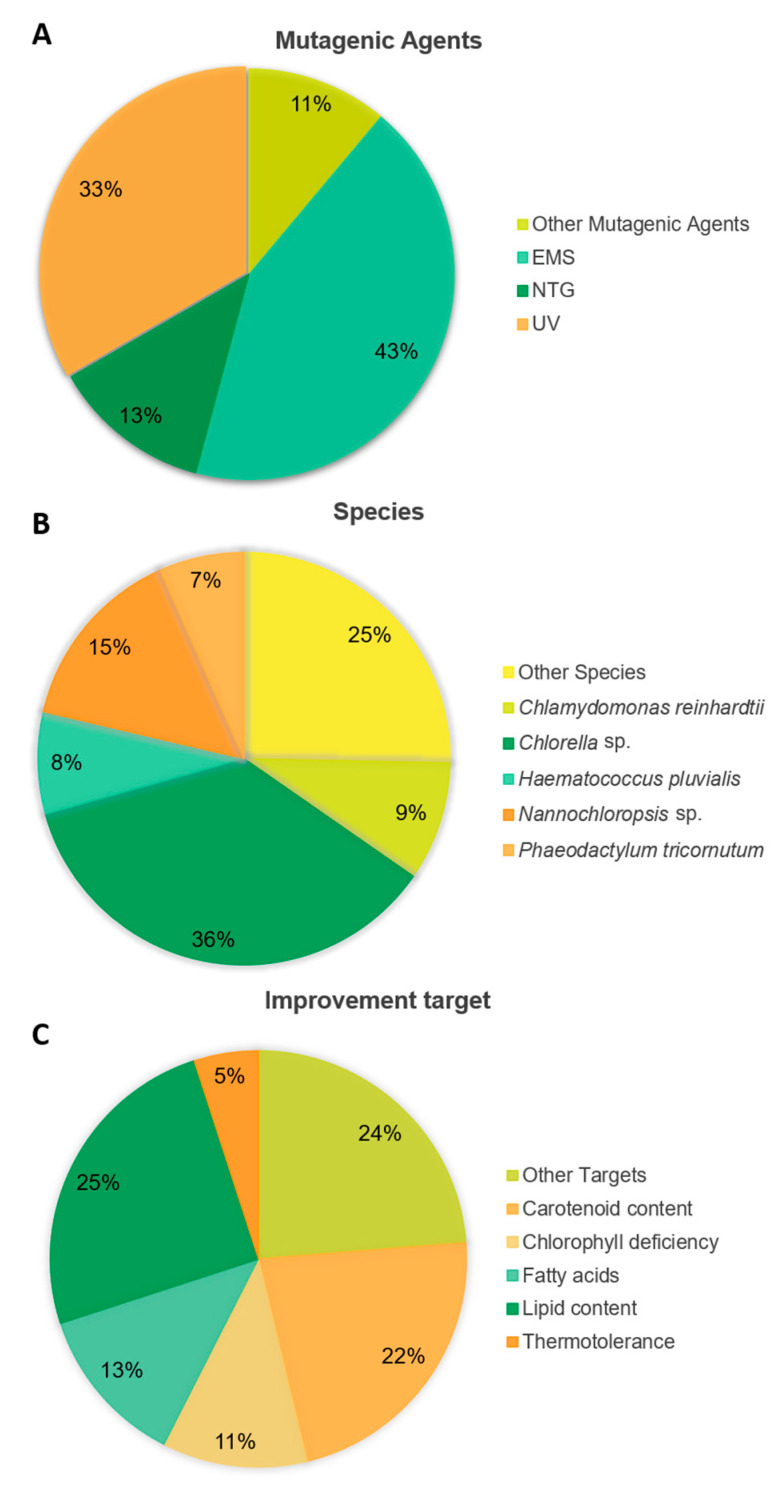
Statistics of random mutagenesis publications (% of reports out of all the examples in this review. (**A**)—Mutagenic agents; (**B**)—Genera and species; (**C**)—Improvement target.

Interestingly, out of the 75 articles published in the literature using random mutagenesis to improve microalgae, EMS was the most widely used mutagenic agent, being the chosen method in 43% of the reports. UV treatment came in second place with 33% of the studies adopting this method (Figure 4A). The most frequently targeted genera were *Chlorella* and *Nannochloropsis* with, respectively, 36 and 15% of studies applying these techniques to these microalgae, most probably a consequence of them being of high commercial interest (Figure 4B). Concerning metabolism as the target for improvement, most research was carried out to enhance lipid and carotenoid productivity, 25 and 22% of the studies, respectively (Figure 4C).

### 2.2. Adaptive Laboratory Evolution

It is common knowledge among the research community that stressful conditions induce microalgae to produce and accumulate different molecules, generally lipids and pigments, that help them survive and cope with environmental stress [6,137,138].

Likewise, adaptive laboratory evolution has been used not only to create hyperproducing strains for industrial cultivation but also to generate more robust and tolerant strains capable of bioremediating toxic compounds, through growing under high concentrations of CO_2_, phosphate, nitrate, or heavy metals, for example [6,8,137].

**Figure 5:**
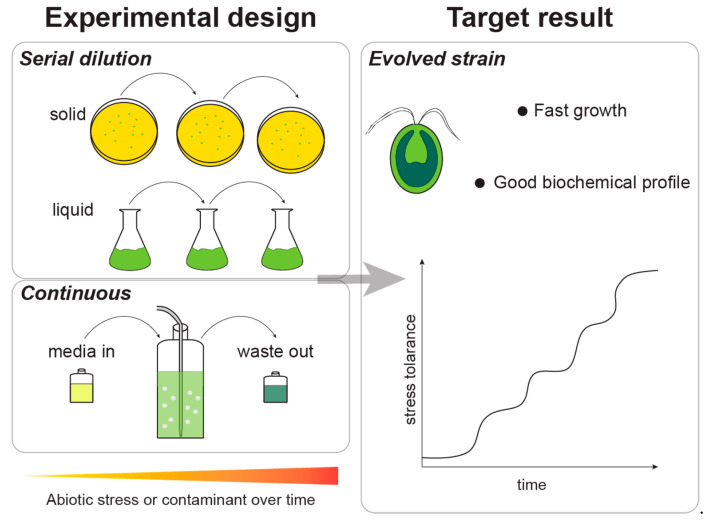
Diagram of adaptive laboratory experiments and expected results. Left—adaptive laboratory evolution experimental designs in batch and continuous mode. The abiotic stress is kept constant or increased, and this leads to the improvement of the culture. Right—after adaptive laboratory evolution, the evolved microalgal strain will be able to tolerate the abiotic stress while maintaining favorable growth parameters and a balanced biochemical profile.

Adaptive laboratory evolution consists of exposing microalgae to specific stress conditions (e.g., high salinity, CO_2_, glucose, or flue gas concentration) during prolonged periods (months or years) to promote the selection of spontaneous mutations that confer an adaptive advantage to the growth conditions (Figure 5). Usually, in adaptive laboratory evolution experiments, the mutations detected have been mapped to stress-induced genes. Under stressful conditions, the stress-induced genes are activated at the expense of housekeeping genes and growth. If the stressful conditions are withdrawn, the stress-induced genes are repressed and the cell resumes its normal activity. The conditions of adaptive laboratory evolution keep the stress constant from one generation to the next and the stress response is kept active, so that any mutation that enables the cell to grow under stressful conditions is likely to be favored. Likewise, each generational cycle improves the original wildtype strain, selecting cells with higher environmental tolerance, and thus, displaying more robust, tailor-made phenotypes [8,78,139].

Adaptive laboratory evolution is an effective strategy to isolate improved strains, since it stimulates the accumulation of beneficial mutations in several genes in parallel, acting in a genome-wide manner, which favors the permanence and stability of the intended alterations [8,78]. Moreover, by inducing stress conditions, the underlying microalgal metabolic mechanisms and responses to environmental stress might be further scrutinized, along with information about genes imparting stress tolerance and the design of novel strains through synthetic biology (experimental evolution) [6]. It is also useful to apply tools, such as FACS, to assist in the selection of the fittest mutants, based on, for example, their cell morphology or pigment content [8]. Adaptive laboratory evolution also allows the study of evolutionary trade-offs, since adaptations that provide better fitness in one environment might lead to maladaptation in another.

However, cells grown in the laboratory might be under evolutionary constraints imposed by lower genetic variation due to the smaller population size as compared to the genetic diversity found in larger microalgal populations present in nature; this can hamper or delay the isolation of mutants with the desired phenotype [51]. As a result, a significant and uncertain number of generations is usually necessary to complete the evolutionary process, which can take from months to years [8,51,78]. This lag in microalgae adaption is also related to their larger genomes and lower growth rates compared to those of bacteria and yeast, and thus, the efficiency of this approach depends on the initial strain chosen for improvement and the stress factors applied [8]. In addition, creating laboratory mutant strains might result in organisms that are unable to thrive on more variable, less predictable environments, such as those of outdoor industrial reactors, since it is hard to mimic such conditions in a laboratorial context [8].

In Figure 5, two different experimental designs of adaptive laboratory experiments are represented: serial dilution (or batch) and continuous. Batch experiments are characterized by the sequential passage of the culture to different media (liquid or solid) under increasing levels of the selective stress condition. Photobioreactors in a continuous operation mode can be used in order to impose an uninterrupted selective pressure to the culture over prolonged periods of time.

In 1997, Rebound and Bell [140] reported the first experiment of adaptive laboratory evolution with microalgae. In this work, they adapted *Chlamydomonas* cell lines to light or dark environments by submitting the populations to different light/dark stress patterns.

As with random mutagenesis, researchers aimed to improve the biochemical profile of the microalgae, namely carotenoids and fatty acids, while maintaining or improving their growth rate (Table 2). Regarding biochemical improvements, Gao et al. [29] have recently reported the isolation of an improved *Tisochrysis lutea* with only two rounds of FACS based selection using fucoxanthin fluorescence, of which fucoxanthin and DHA contents were 3.1 and 1.6-fold higher, respectively (Table 2). Wang et al. [141] managed to increase the EPA content of *Phaeodactylum tricornutum* cells to 139 µg/mg biomass using hyposaline and fulvic acid treatments (Table 2).

Scientists are also focusing on the development of strains able to perform bioremediation, which have to be robust and able to grow in media with high amounts of potentially harmful compounds (e.g., phenol, NOx, SOx, and CO_2_) in order to remove them from the environment. For example, Cheng et al. [78] improved a *Chlorella* strain through an adaptive laboratory evolution of 46 cycles to flue gas, which developed tolerance and became able to grow exposed to the aforementioned pollutants (Table 2). Another example comprising bioremediation is the adaptive evolution reported by Wang et al. [141], in which *Chlorella* sp. was submitted to 31 cycles of exposure to phenol. The improved strain doubled the maximum biomass concentration and removed 100% of phenol from the wastewater (Table 2). Recently, adaptive laboratory evolution was successfully applied to increase the maximum temperature tolerance of microalgae. Barten et al. [144] applied high temperature as a stress factor and were able to increase the maximum temperature that *Picochlorum* sp. tolerated by 1.5 °C (Table 2). This is an important breakthrough, as temperature is one of the factors affecting the production costs of microalgae.

### 2.3. Genetic Engineering

Genetic engineering has been used as a tool to manipulate microalgal genomes in order to create more productive strains with tailor-made features, and to enhance the biosynthesis of valuable target metabolites [2]. Once the target pathways and respective genes are identified, as in forward genetics, genetic engineering and the available molecular tools allow one to insert (“knockin”), delete (“knockout”) or modify a gene in one or more nucleotides [145]. These modifications can lead to the upregulation of a specific gene. Conversely, depending on the mutation generated, it can lead to partial (“knockdown”) or full abrogation (“silencing”) of the expression of the target gene, which can be either permanent or transient [144,145]. Transient gene expression can be either a time-dependent or condition-dependent phenotype, or both. As these genetic modifications might result in the overexpression or silencing of the target gene, which is associated with one or more metabolic pathways, it can be used to manipulate the metabolism in order to produce a completely new metabolite through a gain-of-function mutation (usually through a knockin mutation), or to simply improve the production of a pre-existing metabolite [2]. There are several genetic engineering methods that can be employed to modify microalgae, namely via ZFNs [146], transcription activator-like effector (TALE) nucleases (TALENs) [147], RNAi, *Agrobacterium tumefaciens*-facilitated DNA transformation [148] and/or CRISPR-Cas9 gene editing [149].

**Figure 6:**
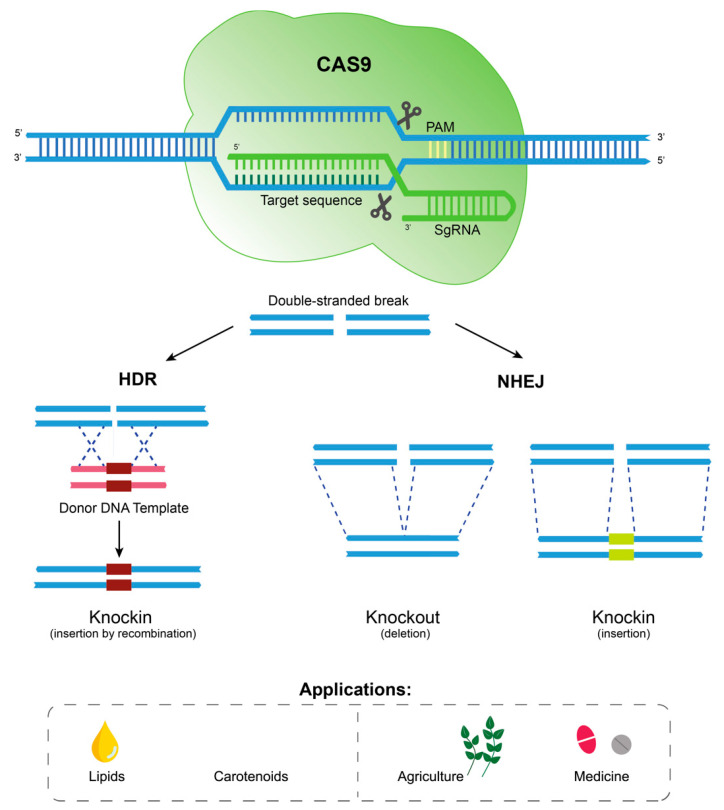
Genome editing using CRISPR-Cas9. The nuclease Cas9, with a custom single-guide RNA (SgRNA), cuts DNA on a specific sequence near a protospacer adjacent motif (PAM), a short sequence recognized by the enzyme downstream of the cleavage site. In the presence of exogenous DNA, homology-directed repair (HDR) can take place, generating a knockin mutant; otherwise, non-homologous end join (NHEJ) repair might occur, so that the ends of the DNA fragments are brought together. The mutant might contain a disrupted target gene (knockout) or an inserted gene or DNA fragment (knockin), which could generate a loss- and/or gain-of-function phenotype. The main applications of this technology are related to improving lipid content and profile, the production of high-value compounds such as carotenoids, the development of tolerance for agroindustrial applications, and the production of recombinant proteins for pharmaceutical and medical applications.

ZFNs, TALENs and CRISPR-Cas9 technologies have been used to edit genomic DNA in order to generate mutants by using the catalytic domain of a DNA-cleaving enzyme, which is then targeted to specific sequences. While ZFNs use zinc finger DNA binding domains to target the nuclease, TALENs contain TALE repeat arrays that can be engineered to bind to specific DNA sequences [148]. CRISPR-Cas9 is a more recent technology that instead uses a guide RNA to target the Cas9 nuclease to cleave at a specific genomic site [150]. All these techniques depend on the generation of double-strand breaks, which can be repaired by non-homologous end-joining (NHEJ), generating indel (insertion/deletion) mutations, or by homology-directed repair (HDR) with the help of donor templates, which can generate precise mutations, down to single-nucleotide mutations and indels (Figure 6) [147,151].

The first microalga to undergo nuclear transformation was *C. reinhardtii* in 1990 using glass bead agitation and electroporation [152]. Other oleaginous species have been the focus of interest in this area, such as *Nannochloropsis* sp., *Phaeodactylum tricornutum, Dunaliella* sp. and *Tetraselmis* sp., as shown in Table 3. Different strategies have been used to enhance lipid production, such as the overexpression of enzymes involved in TAG assembly, PUFA production (EPA and DHA) or NADH biosynthesis [138]. The overexpression of the enzyme diacyl glycerol acyl transferase (DGAT), involved in the last step of TAG synthesis, led to an increment in 79% of EPA and 69% of the neutral lipid contents in *P. tricornutum* and *N. oceanica,* respectively (Table 3) [153,154]. Glycerol-3-phosphate acyltransferase 2 (GPAT) is the first enzyme involved in TAG synthesis and led to a 2.9-fold increase in TAG content when overexpressed in *P. tricornutum* (Table 3) [155]. Moreover, a combination of genetic transformation of *C. reinhardtii* to overexpress acetyl-CoA synthetase or type-2 diacylglycerol acyl-CoA acyltransferase and nitrogen or phosphorus starvation resulted in a 2.4-fold and 2.5-fold increase in TAG content, respectively (Table 3) [156,157]. Gene edition has also been applied to microalgae to obtain higher lipid contents. For example, a CRISPR-Cas9-mediated knockout of NO06G3670 transcription factor was used by Südfeld et al. [158] to enhance lipid accumulation in *N. oceanica* by 40% (Table 3). In addition, Xue et al. [159] obtained a 2.5-fold increase in total lipid concentration in *P. tricornutum* through the overexpression of the malic enzyme (ME), a biocatalyst able to convert malate to pyruvate with the production of NADPH, which plays an important role in lipid biosynthesis [160] (Table 3).

There are several methods used to transfer exogenous DNA into cells, such as electroporation, glass beads, and mediation using biological vectors, such as *Agrobacterium tumefaciens.* The use of the latter vector has been demonstrated to be effective in plants and fungi [165]. When CRISPR-Cas9 technology was applied for the first time to *Chlorella vulgaris,* transformants from the *A. tumefaciens*-mediated method displayed a 46% (*w/w*) higher lipid accumulation (Table 3) [162].

For the purpose of increasing biomass productivity, RuBisCo activase has often been targeted in microalgae to improve the limiting rate of CO_2_ assimilation in photosynthesis. Wei et al. (2017) overexpressed this enzyme in *Nannochloropsis oceanica* and obtained mutants with a growth rate 32% higher compared to that of the wildtype (Table 3) [164].

In addition, the overexpression of enzymes involved in carotenogenesis is also a strategy to increase high-value compounds, such as pigments. The first steps of carotenoid biosynthesis are catalyzed by phytoene desaturase (PDS) and phytoene synthase (PSY). For example, Cordero et al. [161] enhanced violaxanthin and lutein content 2-fold and 2.2-fold, respectively, in *C. reinhardtii*, through the heterologous overexpression of PSY, while Galarza et al. [163] achieved an increase of 67% in astaxanthin content in *H. pluvialis* through PDS overexpression (Table 3).

Gene editing techniques have great potential to create hyperproducing and more productive microalgal strains, since specific genes of interest can be targeted to tailor to the genome to attain the desired traits. Unlike adaptive laboratory evolution and random mutagenesis, genetic engineering can be directed to modify a specific gene or regulatory sequence, whose phenotype can be tested under laboratory conditions [2,78].

Nonetheless, there are several limitations to genome editing, which explains why there are few reports on the successful genetic engineering of microalgae. Firstly, the phenotypes resulting from the mutation of specific genomic sequences must be identified for each species, and the development of consistent genome editing techniques for microalgae are far from a ready-to-use technology [60,78]. Secondly, obtaining genetically stable mutant strains able to thrive under industrial settings and overcome the resistance of transformants to Cas9 toxicity remains a challenge [64]. Finally, some desirable features, such as stress tolerance, are often complex processes that encompass a wide range of genes, making it difficult to achieve significant strain improvement by targeting specific genes via genetic and metabolic engineering [8]. Hence, researchers have resorted to omics (e.g., metabolomics, proteomics, lipidomics and transcriptomics) technologies to predict complex interactions among gene products. This strategy might enhance the outcome of genome editing and overcome the difficulty of improving multigenic traits [2,78].

### 2.4. Regulatory Frameworks on Genetically Modified Organisms (GMOs) Applied to Microalgae

There is a general concern about generating genetically modified organisms by means of the techniques discussed above. In the European Union (EU), a GMO is defined as “an organism, with the exception of human beings, in which the genetic material has been altered in a way that does not occur naturally by mating and/or natural recombination” [32,166]. Using this definition, most microalgal mutants that have not been generated by spontaneous mutations would fall under this definition of a GMO. However, because of their extensive safety track record, and the fact that no foreign genetic material is introduced into the mutant genome, microalgal strains improved by random mutagenesis or adaptive laboratory evolution are exempt from the requirements for those obtained by heterologous DNA transformation/transfection using genetic engineering [6,32,47,166,167]. In the United States, three agencies, U.S. Food and Drug Administration (FDA), U.S. Environmental Protection Agency (EPA), and U.S. Department of Agriculture (USDA), work together to regulate and ensure that GMOs are safe for humans, animals, plants, and the environment on a case-by-case basis [168,169]. These safety measures have been put in place, since the interactions of new strains with natural environments are unknown and there is the possibility of gene flow between species with unpredictable consequences that might unbalance ecosystems, particularly in primary producers at the food web base [2,56]. Higher risks are assigned to herbicide and antibiotic resistance transgenes or genomes with enhanced growth performances that might outcompete microalgal strains in natural environments [32,166]. Accordingly, careful risk assessment and close monitoring should be carried out before providing GMO products to the microalgal market [32,56]. However, as randomly generated mutants or, even better, spontaneous mutants selected by adaptive laboratory evolution are exempt from the requirements of genetically engineered GMOs, the commercialization of the first two types of improved strains appear to be more promising in terms of market demand, as well as existing regulatory frameworks.

## 3. Conclusions

Microalgal strain improvement is essential to provide more productive and robust strains, and to address the current challenges in industrial production. The decision of investing in one strain improvement approach over another should be made in accordance with the improvement target and the intended application. As such, random mutagenesis is a cost- and time-effective strategy to deliver more competent strains for microalgae industry. However, there is still a long way to go concerning the screening and selection of mutants with the desired phenotype. It is important to test and study new metabolic inhibitors and selective pressures to develop selection methods that enable a more effective identification and isolation of different phenotypes. In addition, the potential of high-throughput methods, such as FACS, is still underexploited, since it is limited to a few markers (e.g., pigments autofluorescence and lipid dyes) and, subsequently, few metabolic targets (e.g., lipid and carotenoids contents). These technologies should be further studied to shed light on how the different cell characteristics and fluorescent dyes are related, and the information they can provide about a cell and its compounds. Furthermore, the study of microalgal omics, such as genomics and metabolomics, has an important role in elucidating the regulation of the pathways responsible for the biosynthesis and catabolism of target compounds. This interconnected knowledge will enable the identification, selection and isolation of different factors (e.g., gene products and conditions) that are crucial for the improvement of a specific microalgal strain with a given target phenotype.

## Figures and Tables

**Table 1:** Relevant examples of recent random mutagenesis reports aiming at different targets, indicating the respective mutagenesis method used, species, screening strategy and obtained improvement. An extended version of this table can be found in the Supplementary Materials (Table S1).

Species	Method	Target	Screening	Improvement	References
**Chemical mutagenesis**					
*Chlorella* sp.	EMS 100 mM, 30 min	Lipid content	FACS using BODIPY 505/515 staining	1.4-fold increased lipid content	[109]
*Chlorella* sp.	EMS 100 mM, 60 min	Thermotolerance	Incubation at 40 °C; size	Increase of 1.8-fold at 25 °C and 6.7-fold at 40 °C for growth rate	[104]
*Chlorella* sp.	NTG 5 μg mL^−1^ for 60 min	Alkali tolerance	pH 11.5; size	CO_2_ utilization efficiency	[106]
*Chlorella vulgaris*	EMS 300 mM, 60 min	Chlorophyll deficiency	Color and norflurazon	Up to 99% lower chlorophyll and 60% higher protein content	[11]
*Coelastrum* sp.	EMS 400 mM, 60 min	Carotenoid content	Glufosinate 25 μM and size	2-fold higher astaxanthin content	[110]
*Desmodesmus* sp.	EMS 600–800 mM, 30–60 min	Lipid content	Nile red fluorescence	Increased lipid productivity of up to 74%	[98]
*Nannochloropsis gaditana*	EMS 70 mM, 60 min	Chlorophyll deficiency	In vivo fluorescence imaging	Photosynthetic activity and biomass productivity	[111]
**Physical mutagenesis**					
*Chlamydomonas reinhardtii*	UV, 30 min	Sterols	On 0.1–1.0 mM terbinafine	50% overproduction of sterols and squalene, higher resistance to oxidative stress	[112]
*Chlorella* sp.	Gamma ray, 800 Gy	Lipid content	Nile red fluorescence	Increased lipid content and productivity	[113]
*Phaeodactylum tricornutum*	Heavy-ion irradiation	Carotenoid content	FACS (chlorophyll autofluorescence)	25% higher fucoxanthin content	[88]
*Tetradesmus* (*Scenedesmus) obliquus*	UV 254 nm (40,000 µJ cm^−1^)	Starchless mutants	Iodine vapor staining to screen for starch	41% increased total fatty acid productivity	[114]
**Hybrid mutagenesis**					
*Chlorella vulgaris*	UV 254 nm (0.5–10 min) + EMS 25 mM 60 min	Lipid content	Growth and Nilered staining;	Lipid content and biomass were, respectively, 67% and 35% higher than those of the wildtype	[115]

**Table 2:** Examples of adaptive laboratory evolution reports obtained by different methods. An extended version of this table can be found in the Supplementary Materials (Table S2).

Species	Method	Target	Improvement	References
*Chlorella* sp.	31 cycles under 500 mg/L of phenol	Phenol wastewater removal	100% phenol removal in 7 days; maximum biomass concentration increased 2-fold	[141]
*Chlorella* sp.	46 cycles with flue gas	Tolerance to flue gas	Growth under 10% CO_2_, 200 ppm NOx, and 100 ppm SOx	[78]
*Phaeodactylum tricornutum*	11 cycles, 5 days each, light-induced oxidative stress supplied by LED	Carotenoid content	2-fold higher biomass production and fucoxanthin content	[142]
*Phaeodactylum tricornutum*	35 cycles, 7 days each, of hyposaline treatment	Fatty acid content	EPA content increased up to 139 µg/mg biomass; improved growth	[143]
*Picochlorum* sp.	390 days under temperature stress	Thermotolerance	1.5 °C increase in the maximum tolerable temperature	[144]
*Tisochrysis lutea*	2 rounds of direct evolution + FACS	Carotenoid and fatty acid content	3.1-fold fucoxanthin and 1.6-fold DHA higher productivities	[29]

**Table 3:** Examples of genetic engineering methods in microalgae and the results obtained. An extended version of this table can be found on the Supplementary Materials (Table S3).

Species	Method	Target	Improvement	References
*Chlamydomonas reinhardtii*	Heterologous overexpression of phytoene synthase (PSY)	Carotenoid content	2.0- and 2.2-fold higher in violaxanthin and lutein content	[161]
*Chlamydomonas reinhardtii*	Overexpression of acetyl-CoA synthetase (ACS)	Lipid content	2.4-fold more TAG in N depletion media	[157]
*Chlamydomonas reinhardtii*	Overexpression of type-2 diacylglycerol acyl-CoA acyltransferase (DGTT4)	Lipid content	2.5-fold increased TAG content in P depletion media	[156]
*Chlorella vulgaris*	Heterologous overexpression of mGFP	Lipid content	46% (*w*/*w*) higher lipid accumulation	[162]
*Haematococcus pluvialis*	Overexpression of phytoene desaturase (PDS) gene	Carotenoid content	67% increase in astaxanthin accumulation	[163]
*Nannochloropsis oceanica*	Knockout of NO06G03670	Lipid content	Increase in neutral lipids content by 40%	[158]
*Nannochloropsis oceanica*	Overexpression of RuBisCO activase	Growth productivity	Growth rate and photosynthesis increase by 32 and 28%, respectively, induced under low level of CO_2_	[164]
*Nannochloropsis oceanica*	Overexpression of type 2 diacylglycerol acyltransferase (DGAT)	Lipid content	69% increase in neutral lipid content	[154]
*Phaeodactylum tricornutum*	Overexpression of glycerol-3-phosphate acyltransferase 2 (GPAT2)	Lipid content	2.9-fold increase in TAG content	[155]
*Phaeodactylum tricornutum*	Overexpression of malic enzyme	Lipid content	2.5-fold increase in total lipid content	[159]
*Phaeodactylum tricornutum*	Overexpression of type 2 DGAT	Lipid content	76% increase in EPA content	[153]

## Data Availability

Additional data will be provided on request.

## Supplementary Materials

The following are available online at https://www.mdpi.com/article/10.3390/md20070440/s1. Table S1: Extended version of Table 1: examples of random mutagenesis reports, where the respective mutagenesis method used, target, species, screening strategy, and obtained improvement is referred; Table S2: Extended version of Table 2: examples of adaptive laboratory evolution reports aiming at different targets, where the respective method used, target, species, screening strategy, and obtained improvement is referred; Table S3: Extended version of Table 3: examples of genetic engineering reports, where the respective method used, target, species, and obtained improvement is referred.
